# The Instruments Used to Assess Health Literacy and Pharmacotherapy Literacy of Diabetes Mellitus Type 2 Patients: A Scoping Review

**DOI:** 10.3389/fpubh.2021.747807

**Published:** 2021-09-27

**Authors:** Marija Levic, Natasa Bogavac-Stanojevic, Dusanka Krajnovic

**Affiliations:** Faculty of Pharmacy, University of Belgrade, Belgrade, Serbia

**Keywords:** health literacy, medication literacy, measurement tool, assessment, patient, chronic disease, healthcare system

## Abstract

**Background:** Patients with chronic diseases, like diabetes need to continuously perform tasks associated with self-management especially with medications they use. It is shown that the patients with diabetes with limited HL and PTHL cannot read medication labels correctly, may misuse their medications, spend much more on therapy and generally have difficulties in understanding printed care instructions and perceiving health advice and warnings. There has been an increasing demand for valid and reliable instruments for HL and PTHL assessment in this population. This review aims to search and critically discuss instruments used to assess HL and PTHL in people with type 2 diabetes and propose their use in different settings.

**Methods:** Authors conducted a comprehensive, electronic search of original studies using a structured approach of the Scopus and PubMed databases, during November and the first 2 weeks of December 2020 to find relevant papers. The review was conducted in accordance with the Cochrane guidelines and the reporting was based on the PRISMA-ScR. The comparison of instruments was made by utilizing a comparison model related to their structure, measurement scope, range, psychometric properties, validation, strengths, and limitations.

**Results:** The final number of included studies was 24, extracting the following identified instruments: Korean Functional Test HL, NVS, FCCHL, HLS-EU-47, TOFLHA, S-TOFHLA, REALM-R, 3-brief SQ, REALM, HLQ and DNT-15. In all, FCCHL and 3-brief SQ are shown with the broadest measurement scopes. They are quick, easy, and inexpensive for administration. FCCHL can be considered the most useful and comprehensive instrument to screen for inadequate HL. The limitation is that the English version is not validated. Three-brief SQ has many advantages in comparison to other instruments, including that it is less likely to cause anxiety and shame. These instruments can be considered the best for measuring functional HL in patients with diabetes mellitus type 2 and other chronic diseases. PTHL instruments (REALM and DNT-15) did not find the best application in this population.

**Conclusions:** The future research should be directed in validation of the FCCHL in English and establishing of the structural validity of this questionnaire. Developing a specific PTHL questionnaire for this population will be of great help in management of their disease.

## Introduction

The main goal of today's healthcare system is to promote and maintain good health and at the same time enable people to take care of their health. People are also expected to be more responsible for their health and participate more in decision-making related to their health (1–3). When we talk about responsibility for our health, we must consider different assumptions. Efforts should be focused on a person's ability to cope and take responsibility for their health (3). The Patients' Rights Act from 1999 states that the manner of participation in healthcare decisions should be adapted to each person's ability to give and receive health information. These abilities are related to health literacy (HL) (4).

To understand and use health information to make health decisions, adequate HL is needed (4). In a report by the World Health Organization (WHO), HL is one of the most important determinants of health. HL can be considered necessary to control and monitor one's health (5). Several studies have shown that most people have limited HL (6–12). Also, people with low HL are more likely to be with poorer health, more prone to complications, and have a higher mortality rate than people with high HL (5).

Healthcare professionals should consider that individuals possess different levels of HL. Therefore, knowledge about HL of people is necessary to adapt better health professionals' communication with different target groups, which would make the information more beneficial for the individual by enabling them to participate in health decisions and take responsibility for their health (13).

Optimizing health communication can prevent misunderstandings and other complications, thus the quality of care and patient safety would be improved (13). In order to meet expectations such as increased participation and responsibility for one's health, it is necessary to consider HL in individuals and the general population. The purpose of today's public health policy is to create conditions for educating people to be able to take control of their health and control it (3). Therefore, measuring HL in different populations would provide essential knowledge that would be used to improve health communication, and thus the ability of individuals to control their health. However, the validity and reliability of individual HL instruments have not been adequately established (14) and only a few HL instruments were validated using modern test theory, such as Rasch modeling (15, 16).

### Health Literacy

Adequate health literacy is crucial for patients to make optimal choices for their health and medications management. Additionally, successful health communication presupposes certain levels of competence of both the healthcare professional and the patients and is adapted to the HL of the individuals (5).

“HL” as a recognized term came into use around 1974, but only became a [Medical Subject Headings (MeSH)] term in MEDLINE in 2006. A systematic review of Sørensen et al. (17) discovered 17 definitions and 12 conceptual models of HL. Based on all the offered, one overall definition was obtained, which reads: “HL is linked to literacy and entails people's knowledge, motivation and competencies to access, understand, appraise, and apply health information in order to make judgments and take decisions in everyday life concerning healthcare, disease prevention and health promotion to maintain or improve quality of life during the life course.” There is currently no consensus on defining HL, which means that different approaches to this term are used in various research environments (17–20). An additional concept of understanding becomes a problem when it comes to assessing and measuring HL and then comparing these results between different studies (16). The presence of different definitions is probably due to the fact that the concept has been developed in different parts of the world, with varying abilities and skills considered necessary to deal with health information in each specific context (21).

In the twentieth century, reading and writing were sufficient to use information obtained from health professionals. However, with increasing expectations of active participation in health decisions, increasing responsibility for one's health, and digital development in health information, additional skills are needed to handle health information. First, the need for reading has increased, and the skills to apply and critically evaluate health information from various sources are essential. HL combines a set of skills or abilities, while on the other hand it depends on the requirements to which the individual is exposed. The impact of technological development has also increased, which will affect the definition and understanding of HL in the future (22).

### Pharmacotherapy Literacy (PTHL)

Patients with chronic diseases need to continuously perform tasks associated with self-care and self-management of their medications. When taking medicines, they constantly need abilities related to various domains of HL, so HL brings together many concepts that are associated with patient's pharmacotherapy. Whether they rely on information in printed materials or verbal instruction patients with chronic conditions need to have adequate HL related to medications as critical for managing their conditions. Due to the complexity of the various procedures required for the adequate use of medications, the concept of PTHL was introduced.

King and colleagues, in consultation with the academy and pharmacists, formulated PTHL as: “*An individual's capacity to obtain, evaluate, calculate, and comprehend basic information about pharmacotherapy and pharmacy related services necessary to make appropriate medication-related decisions, regardless of the mode of content delivery (e.g., written, oral, visual images and symbols)*” (23). This definition was updated by adding to reduce thereby the risk of poor pharmacotherapy outcomes (24).

### HL and PTHL in Persons With Type 2 Diabetes Mellitus

Diabetes is one of the most common chronic non-communicable diseases and is a major public health problem. In 2019, the International Diabetes Federation estimated that 463 million adults worldwide have diabetes and that this number is expected to increase to 700 million by 2,045. The cause of this disease is multifactorial, but it is associated with unhealthy lifestyles such as physical inactivity and poor diet. It is assumed that between 30 and 80% of people with type 2 diabetes are still undiagnosed. Complications such as diabetic nephropathy and neuropathy may occur at a later diagnosis of disease (25). Despite advances in therapy and the availability of clinical practice guides, only 30% of patients manage to achieve the target values of glycemia, cholesterol and blood pressure. The fact is that patients perform 95% of diabetes care on their own (26).

Type 2 diabetes is more common than type 1, so 90% of all diabetes is type 2 diabetes. It most often occurs in middle age and in the elderly. It is closely related to lifestyle and health habits, with being overweight and obese being risk factors. Hereditary factors can also influence the risk of developing this disease. Therapy includes weight loss, diet and therapy with drugs that lower blood glucose levels. Affected people are advised to give up cigarettes and reduce alcohol intake to prevent the appearance of cardiovascular diseases. Living with this disease requires changes in health behavior, self-control, and a lot of care (27–29). Since living with type 2 diabetes requires a lot from people with the disease, these persons must be informed about therapy, diet and other health behaviours, which require adequate HL and PTHL. Several international studies have shown that reading and understanding the guidelines for modern diabetes medications, applying appropriate dietary restrictions, and gaining insight into the physiological processes involved in the disease can be a major challenge for an individual (30, 31). The performing of diabetes self-management tasks frequently involves abilities, such as taking medications at the right time, interpreting blood glucose levels and calculating insulin doses.

A recent review of HL and health outcomes in patients with type 2 diabetes concludes that there is strong evidence to suggest a positive correlation between HL and diabetes knowledge (32). It is also considered that there is sufficient evidence to support a link between HL and self-care (33). On the other hand, the evidence of a link between HL and clinical indicators was inconsistent (34).

Some primary studies that looked at the level of HL in patients with type 2 diabetes found that a small number of these patients had adequate levels of HL (35–39).

Patients with diabetes and limited HL and PTHL often cannot read drug medication labels correctly, may misuse their medication, do not understand the meaning of consent forms, and generally have difficulty understanding printed care instructions and reading health advice and warnings (40–43). For this reason, is very important to assess their PTHL and in case of needs perform adequate training in order to improve control of their disease and pharmacotherapy management.

These patients also have poorer communication with doctors and participate less in making health decisions (4). Patients who are diagnosed have to make health decisions daily and must also perform complex self-care activities to keep the disease under control. Interventions in upgrading HL education and intensive diabetes-related education have shown good results in patients with limited HL to improve diabetes outcomes (44, 45). People with type 2 diabetes should undergo diabetes education programs at the time of diagnosis and then once a year. This education aims to enable individuals to participate in informed decision-making and disease control, all with the aim of better outcomes in treatment of this disease, improvement of glycemic control, prevention of complications and comorbidities, and improvement of quality of life (46). Education for diabetics should be evidence-based, have specific goals, and be tailored to the needs of individuals. However, the effectiveness of this education depends on individuals, i.e., characteristics such as age, gender, ethnicity, level of HL, ability to take care of themselves, all of which should be taken into account when planning and implementing this type of education (27, 47). In this way, they will be able to understand and use the information they receive to maintain health and control diabetes in everyday life (47).

### Instrument Development

In the past 25 years, numerous instruments have been developed to measure HL and PTHL in various contexts (14, 24, 48). These instruments significantly differ in structure, measurement, range, and psychometric properties. The diversity of instruments has led to inconsistencies in measurement with the complexity of interpreting the results and choosing suitable instruments for new studies. Several studies have examined the variation through the range of the most used HL instruments (49, 50). Such variations can come from the fact that the instruments measure different conceptual dimensions of HL. However, it may be difficult for health professionals or researchers to choose the best instrument when they are unfamiliar with measurement properties. Another very important consideration in selecting a HL instrument is its mode of administration. In a subjective instrument, individuals self-report their perceived levels of literacy skills, such as using Likert scales. In contrast, an objective instrument is the interviewer-administrated instrument and assesses the ability to process information by asking respondents to answer specific questions, such as about the time to take the next medication. A subjective instrument requires less cognitive effort in responding to questions, whereas an objective instrument assesses health numeracy more accurately. A self-administrated instrument can be more practical in a very busy clinical settings, than interviewer-administered instrument. Sometimes, the interviewer-administrated instrument may result in discomfort or embarrassment for patients who have a low HL.

Although the instruments were used in several populations, due to the complexity of the tasks and skills that people with type 2 diabetes require, their usefulness and applicability for this population remain challenging. With the growing interest in this construct, there has been an increasing demand for valid and reliable instruments for estimating HL and PTHL.

A systematic review of measurement properties has been designed for providing a comprehensive overview of the available instruments and identifying the best currently available instrument for general population (51). In the previous reviews of HL instruments methodological limitations were identified, such as being descriptive rather than systematic reviews, or lacking quality assessment or data synthesis (14, 52, 53). To address these limitations, a scoping review was conducted to systematically collect the literacy instruments used in people with type 2 diabetes and meet needs for understanding the characteristics, scope of measurements, and their applicability in this population.

## Methodological Study Design

### Aim

This study aims to analyze instruments used in patients with type 2 diabetes mellites for measuring HL and PTHL, in relation to their characteristics (measurement scope, structure, domains, method of scoring), validation, strengths, limitations and accordingly to propose applicability of these instruments in clinical and research settings. This work can be useful as an inventory for researchers and practitioners who are seeking to identify validated measurement instruments in patients with type 2 diabetes mellitus and other chronic diseases that are fitting the best for their research and practice.

### Materials and Methods

Authors built a search strategy by using the PICOS questionnaire. During November and the first 2 weeks of December 2020, a systematic search of the Scopus and PubMed databases was performed in search of peer-reviewed literature of patients with type 2 diabetes mellitus. The protocol of this systematic review (including the article identification strategy and data collection form, etc.) mainly referred to the Cochrane Handbook for Systematic Reviews of Interventions (54) and the reporting of this systematic review was based on the Preferred Reporting Items for Systematic Reviews and Meta-analyses (PRISMA) statement (55).

#### Search Strategy

The keywords used were: “medication literacy,” “measurement tool,” “assessment,” “patient,” “chronic diseases” and “healthcare system.” A search of the terms above found that the number of articles specifically mentioning PTHL was limited, so the search was extended to articles mentioning “health literacy” combined with patients with type 2 diabetes.

In the articles obtained by this search, the references were manually checked to identify additional articles of importance for the work.

#### Study Screening and Selection

All original articles in English are taken into consideration, which meet the below criteria. Duplicates have been excluded. The evaluation of studies regarding the inclusion and exclusion criteria was performed by a pair of independent reviewers (ML and DK): (1) review of titles and abstracts of articles related to the topic (2) review of complete articles was done which examined the HL and PTHL of patients with type 2 diabetes and had the original results of the health and pharmacotherapeutic literacy of patients with type 2 diabetes mellitus conducted through appropriate questionnaires. After cross-checking, a third reviewer (NBS) resolved cases of disagreement.

#### Inclusion and Exclusion Criteria

Peer-reviewed papers with cross-sectional studies, longitudinal studies and cohorts were included if they were: published between the period 2006–2021, written in English, involved patients with type 2 diabetes and papers in which HL and PTHL were examined in patients with type 2 diabetes.

The exclusion criteria were: reviews, case reports, book chapters, letters, editorials, studies that did not address HL and PTHL among patients with type 2 diabetes, studies that did not use the questionnaire for assessing literacy, studies not available or not published in English.

The inclusion and exclusion criteria are presented in Table 1.

**Table 1:** Inclusion and exclusion criteria.

	**Inclusion criteria**	**Exclusion criteria**
Population	Patients with type 2 diabetes mellitus	Patients with other diseases
Type of the study	Cross-sectional, longitudinal, Cohort	Reviews, case reports, book chapters, letters, editorials
Instruments	Using the questionnaire for accessing health/and pharmacotherapy literacy	Works that did not use the questionnaire for accessing literacy (health/and pharmacotherapy)
Language	English	Studies not available or not published in English
Other	Availability of abstract The full text available Year of publication > 2006	Unavailability of abstract The full text not available Year of publication <2006

#### Data Extraction and Synthesis of Results

Data extraction was performed independently by the authors. They extracted different characteristics from each publication, such as (i) publication information: author and year; (ii) study characteristics: country, setting, population, number of participants and results in terms of HL and PTHL (iii) HL and PTHL instruments: name, dimensions, number of items, purpose, target population, administration mode, validation process, scoring, cut-off points, strengths and limitations.

The studies were grouped according to instruments used for the measurement of literacy: HL and PTHL. A descriptive synthesis of the identified studies was performed, and variables described in the synthesis include number of participants, setting, country, population and results. Formal analysis of the results was a descriptive synthesis of the identified instruments from selected studies to determine instruments key characteristics including identifying domains, length of tool (number of questions/items/domains), time for completion, format, and psychometric properties.

#### Critical Appraisal

At the time of our research, there were no accepted quality assessment instruments for cross-sectional studies (56), authors decided to choose a relatively widely used scale, the Agency for Healthcare Research and Quality scale (AHRQ scale) with 11 items, each of which was answered with “yes,” “no” and “unclear.” Two researchers (DK and ML) independently evaluated the quality of the included articles using the AHRQ scale. Any disagreements after cross-checking were resolved by discussions between the two researchers with the final decisions of the third researcher (NBS). If the answer was “no,” “unclear” or “not applicable,” the item was given a score of “0”; if the answer was “yes,” the item was scored as “1.” The quality assessments of the articles were classified as follows: low quality = 0–3, medium quality = 4–7, high quality = 8–11 (57).

The quality assessment of the identified studies is presented in Table 2. The majority of them were classified with medium quality and one fulfilled the criteria for high.

**Table 2:** Critical appraisal of identified studies: quality assessment.

**References**	**Questions**											**Quality of studies**
	**Q1**	**Q2**	**Q3**	**Q4**	**Q5**	**Q6**	**Q7**	**Q8**	**Q9**	**Q10**	**Q11**	
Klinovszky et al. (58)	1	1	1	1	0	0	0	0	0	1	0	5 medium
Hashim et al. (59)	1	1	1	1	0	0	0	0	0	1	0	5 medium
Gomes et al. (60)	1	1	1	0	0	1	0	0	0	1	0	5 medium
Finbråten (61)	1	0	1	1	0	1	0	0	1	1	0	6 medium
Tseng et al. (62)	1	0	0	1	0	0	0	0	0	1	0	3 low
Friis et al. (63)	1	0	1	1	0	0	0	0	0	1	0	4 medium
Sayah et al. (64)	1	1	1	1	0	1	0	1	1	1	0	8 high
Thurston et al. (65)	1	1	1	1	0	0	0	1	0	1	0	6 medium
Mantwill et al. (66)	1	1	1	1	0	0	0	1	0	1	0	6 medium
van der Heide et al. (67)	1	0	1	1	0	1	0	0	1	1	0	6 medium
Sayah et al. (68)	1	1	1	1	0	0	0	0	0	1	0	5 medium
Bauer et al. (69)	1	0	1	1	0	0	1	0	0	1	0	5 medium
Coffman et al. (70)	1	1	1	1	0	0	0	0	0	1	0	5 medium
McCleary-Jones, (71)	1	1	1	1	0	0	1	0	0	1	0	6 medium
Glasgow et al. (72)	1	1	1	1	0	1	0	0	0	1	0	6 medium
Bains et al. (73)	1	1	1	1	0	1	0	0	0	0	0	5 medium
Mancuso, (74)	1	1	1	1	0	0	0	0	0	1	0	5 medium
Osborn et al. (37)	1	1	1	1	1	1	0	1	0	1	0	8 high
Sarkar et al. (75)	1	1	1	1	0	0	1	1	0	1	0	7 medium
Mbaezue et al. (76)	1	1	1	1	0	0	0	0	0	1	0	5 medium
Kim, (77)	1	1	1	1	0	0	0	0	0	1	0	5 medium
Ishikawa et al. (78)	1	0	1	1	1	0	1	0	0	1	0	6 medium
Gebretsadik et al. (79)	1	1	1	1	0	0	1	1	0	1	0	7 medium
Morris et al. (80)	1	0	1	0	0	1	0	0	0	1	0	4 medium

*Q1: Define the source of information (survey, record review) Q2: List inclusion and exclusion criteria for exposed and unexposed subjects (cases and controls) or refer to previous publications Q3: Indicate time period used for identifying patients Q4: Indicate whether or not subjects were consecutive if not population-based Q5: Indicate if evaluators of subjective components of study were masked to other aspects of the status of the participants Q6: Describe any assessments undertaken for quality assurance purposes (e.g., test/retest of primary outcome measurements) Q7: Explain any patient exclusions from analysis Q8: Describe how confounding was assessed and/or controlled Q9: If applicable, explain how missing data were handled in the analysis Q10: Summarize patient response rates and completeness of data collection Q11: Clarify what follow-up, if any, was expected and the percentage of patients for which incomplete data or follow-up was obtained*.

## Results

### Study Screening and Selection

**Figure 1:**
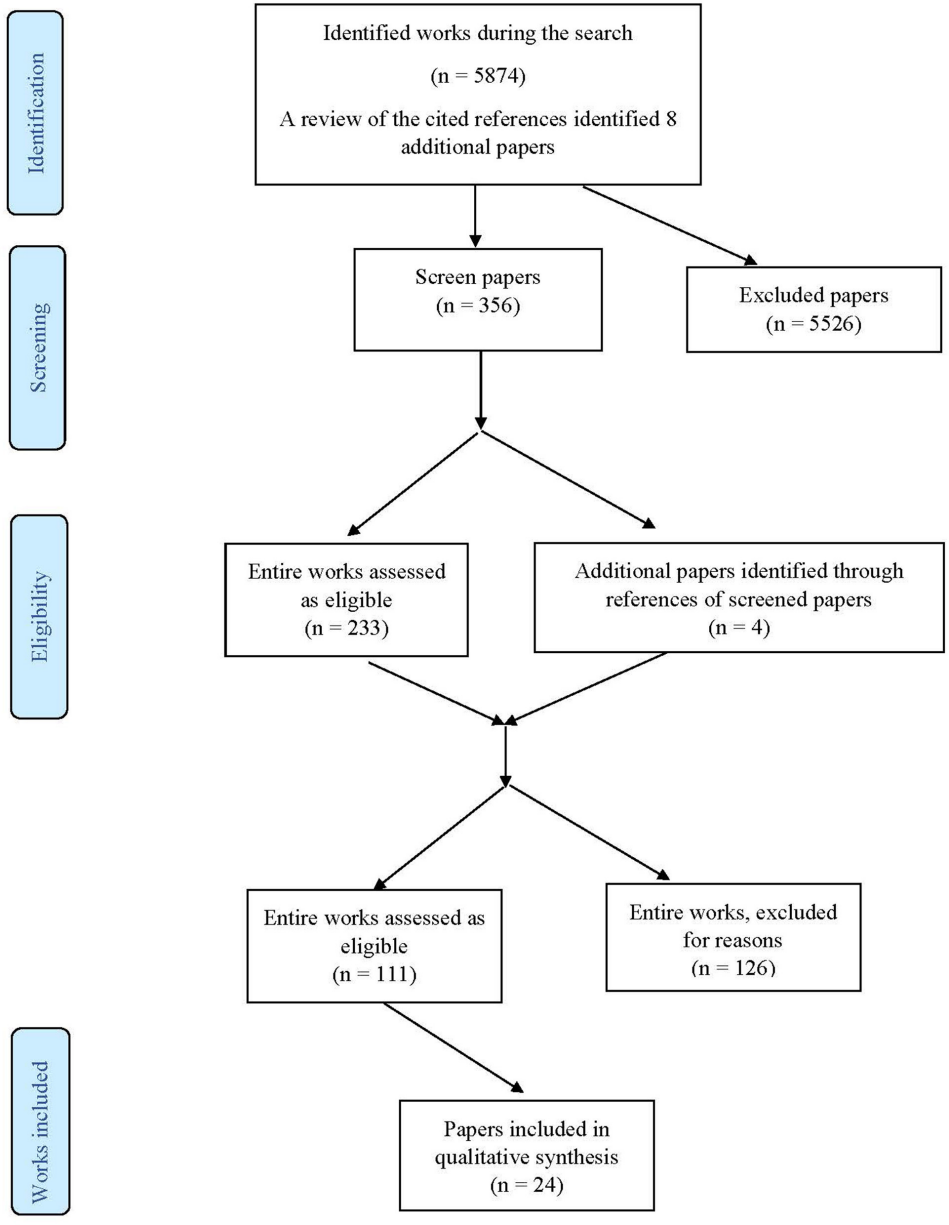
Flow diagram for work extraction.

The PRISMA flow chart in Figure 1 summarises the results of the search process. 1. A search obtained 5,874 potentially relevant studies, while 8 were found by reference review. A cursory review of the content of the papers left 356 papers for further evaluation. Further exclusion (after reading the abstracts and methodology) was based on the principle of excluding papers that mentioned the instrument for assessing HL and PTHL, but without analyzing its structure, and excluding papers that are duplicates (same author, same instrument) left 111 articles for full reading, of which the final number of included studies was 24.

The comparison method was used to compare the instrument in terms of their structure (number and type of questions), the way of reporting, version, purpose, place where they were developed, target population, the person who developed it, year of publication, scoring, heath literacy domains, time and way for administration, measurement scope, validation, strengths, and limitations.

A thorough analysis has been presented in the Tables 3–5. Tables 3, 4 present the instruments used in the studies to assess HL and PHTL, their basic characteristics: domains, methods of assignment, structure and method of scoring. Table 5 shows the psychometric characteristics of the instruments, strengths and limitations.

**Table 3:** Basic characteristics of the included studies for health literacy.

**Name of the instrument**	**Objective/ Subjective (Self-reported)**	**Version**	**Purpose of the instrument**	**Where it was developed/target population**	**Who developed the instrument/ Year of publication/ reference**	**Version in language**
3-brief SQ	Self-reported	Short version	Self-report of confidence in HL skills	USA/Adults	Chew et al. (49)	English
KHLS	Objective	Original	Screening test for limited HP for older Korean adults	Korea/Older Korean adults	Lee et al. (81)	Korean
FCCHL	Objective	Original	Self-report of HL skills	Japan/Adults	Ishikawa et al. (78)	Japanese
HLS-EU-Q47	Self-reported	Original	Questionnaire to assess the relation between abilities, system demands, and decision making	Greece, Ireland, and the Netherlands/15+ years	Sørensen et al. (82)	Available in more than 10 languages
NVS	Objective	Original	Information presented on a nutrition label for reading, comprehension, and numeracy	USA/Adults	Weiss et al. (83)	Spanish, Japanese, Dutch, Turkish, Chinese, Croatian, Italian and Brazilian
TOFHLA	Objective	Original	Close style reading comprehension of health-related content	USA/Adults	Parker et al. (84)	English, Spanish, Chinese, French, German and Italian
S-TOFHLA	Objective	Short version	To measure patients' ability to read and understand health-related materials	USA/Adults	Baker et al. (85)	English, Spanish, French, German and Italian
REALM	Objective	Original	A rapid screening tool to help physicians to identify patients with reading disabilities and assess reading levels	USA/Adults	Davis et al. (86)	English
REALM-R	Objective	Revised form	Word recognition and pronunciation test	USA/Adults	Bass et al. (87)	English
HLQ	Self-reported	Original	Survey items for measuring health literacy of individuals	Australia/Adults	Osborne et al. (88)	English
DNT-15	Objective	Short version	Reading recognition, spelling, and arithmetic computation	USA/Adults	Huizinga et al. (89)	English, Spanish

*3-brief SQ, 3 brief screening questions; KHLS, Korean functional Health Literacy Test; FCCHL, Functional, Communicative and Critical Health Literacy scale; HLS-EU-Q4 7, Health Literacy Survey European Questionnaire47; NVS, Newest Vital Sign; TOFHLA, Test of Functional Health Literacy in Adults; s-TOFHLA, Test of Functional Health Literacy in Adults–Short Form; REALM, Rapid Estimate of Adult Literacy in Medicine; REALM-R, Rapid Estimate of Adult Literacy in Medicine–Revised; HLQ, Health Literacy Questionnaire; DNT, Diabetes Numeracy Test*.

**Table 4:** Basic characteristics of the included studies for pharmacotherapy literacy.

**Name of the instrument**	**Scoring**	**Heath literacy domains**	**Time for administration**	**Measurement scope**	**Total number of items**	**Who administers the tool**
3-brief SQ	Values of 0, 1, 2, 3, and 4 are assigned to each response option for each question; Score ranges from 0 to 12; High scores = high HL skills; Low scores = low HL skills	Functional and critical HL	1–2 min	Reading and understanding health information, completing medical and health care form	3	Self-administered or researcher administered
KHLS	Sum score	Functional HL and numeracy	15–20 min	Literacy, interaction, comprehension, numeracy, information seeking, application and decision making	24	Researcher administered
FCCHL	Each item is scored on a 4-point scale ranging from 1 (*never*) to 4 (*often*); The scores of the items are summed up and divided by the number of the items in the scale. Higher scores indicate higher HL level	Functional, communicative, and critical HL	5–6 min	Reading, understanding applying health information and communication with health care providers	14	Self-administered or clinician/ researcher administered
HLS-EU-Q47	The 47 items are adapted to a 50-point scale: 0–25: inadequate health literacy. 26–33: problematic health literacy; 33–42: sufficient health literacy; 42–50: excellent HL	Functional HL	12–15 min	Interaction, comprehension, information seeking, application, decision making and evaluation	47	Self-administered
NVS	Each item answered correctly is given a score of 1. Scores range: 1–6 (score <4 = limited HL)	Functional, critical HL and numeracy	3 min	Reading, understanding, and applying health information	6	Self-administered
TOFHLA	Scores range 0–100: <60 = inadequate HP; 60–75 = marginal HL; >75 = adequate HP	Functional HL and numeracy	22 min	Reading, understanding applying health information and communication with health care providers	50	Self-administered
S-TOFHLA	Scores range 0–36: 0–16 = inadequate HL; 17–22 = marginal HL; 23–36 = adequate HL	Functional HL	7 min	Applying health information and communication with health care providers	36	Self-administered
REALM	Grade is assigned based on total score that ranges from 0 to 66: 0–18 = ≤ 3rd grade, 19–44 = 4th-6th grade, 45–60 = 7th-8th grade; 61–66 = ≥9th grade	Functional HL	<3 min	Reading and understanding health information	66 words	Researcher administered
REALM-R	Grade is assigned based on total score that ranges from 0 to 8. Score ≤ 6 corresponds to 6th grade and indicates poor HL	Functional HL	<2 min	Reading and understanding health information	8 words	Researcher administered
HLQ	Independent scales that measure proportions of nine competencies for HL	Functional and critical HL	5–10 min	Interaction, comprehension, information seeking, application and decision making	44	Self administered
DNT-15	Items are scored as binary outcomes: correct or incorrect. Scores are reported as percent correct (with a possible range of 0% to 100%)	Functional HL and numeracy	10–15 min	Reading and understanding health information	15	Researcher administrated or self administered

**Table 5:** Instruments' validation, sterngts and limitations.

**Name of the tool**	**Validation**	**Strengths**	**Limitations**
3-brief SQ	Tested against STOFHLA, items AUROC curve ranged from 0.76 to 0.87 (95% CI). The grouped items, including a fourth item about verbal information, (BRIEF), demonstrated an AUROC curve of 0.79 (95% CI) for identifying inadequate skills. Correlations as grouped items against S-TOFHLA (0.42) and REALM (0.40) in multiple demonstrating moderate correlation.	The instrument is validated in several diverse sample populations. It is quick, easy, and inexpensive for administration. Functional domains associated with inadequate HL are assessed. Less likely to induce anxiety and shame.	Methods typically relied on convenience samples. Self-assessment has potential for self-report bias.
KHLS	The overall fit of the two-factor model of the scale was assessed by root mean square error of approximation (0.039), indicating a good fit (criterion 0.05 or less) with an internal consistency of 0.89.	Measure uses questionnaire format containing short passages, pictures, and graphs with multiple-choice answer format, providing a skills-based approach to measurement. Authors used factor analysis methods for development.	No concurrent validity assessed due to lack of a comparative instrument. 10% of study participants needed assistance from interviewers.
FCCHL	Reliability: Cronbach's α: Overall scale: 0.78; Functional domain: 0.84; Communicative domain: 0.77; Critical domain: 0.65	This scale includes three levels of HL, each of which might have different effects on patient outcomes. It is proved to be easy to administer in a clinical setting.	HL was measured based on a self-reported questionnaire. Individuals with reading problems are often ashamed and hide their inability to read, which might have led to an overestimation of the HL levels. Not validated in English.
HLS-EU-Q47	Correlated with NVS (0.25). A multivariate linear regression model with the total sample measured the relation between social variables and health literacy yielding an adjusted R2L17.4%, pL.00. Financial deprivation was the strongest predictor of health literacy.	Available in many languages. Comprehensive, conceptual based measure of most dimensions of health literacy.	Self-assessment has potential for self-report bias. Length of assessment increases response burden.
NVS	Reliability of Cronbach's alpha in English (0.76) and on Spanish (0.69) and correlates with TOFHLA (0.49). The AUROC curve is 0.88 for the English version and 0.72 for the Spanish version.	The NVS test is suitable for rapid assessment of low HL.	Validation sample did not fully represent a demographically diverse population. Test format might intimidate respondents.
TOFHLA	Reliability: Cronbach's α: 0.98; Validity: 0.84 (with REALM),.0.74 (with WRAT-R)	It has been validated in several diverse populations. Available in different languages.	Long version is time consuming. This version is more useful as a research tool than a clinical screening tool.
S-TOFHLA	Reliability: Cronbach's α: 0.98; Validity: 0.91 (with TOFHLA), 0.80 (with REALM)	Short version is available. It has been validated in several diverse populations.	It may not capture an individuals' HL in the dimension of numeracy.
REALM	Correlated with WRAT-R2 (*r* = 0.82) WRAT-R3, (0.88); SORT-R, (0.95, 0.96); PIAT-R, (0.94, 0.97); TOHFLA, (0.30, 0.84). Test-retest correlation: test-retest reliability 0.98 and 0.99.	It is quick and easy for administration. It is short, can be easily administered with minimal training, and it's strongly correlated with standardized literacy assessments.	Only measures one dimension of HL. Limited to the ability to pronounce words without being able to measure the patient's ability to understand the instructions on labeling of prescribed drug.
REALM-R	Reliability: Cronbach's α: 0.91. Validity:0.72 (with REALM), 0.64 (with WRAT-R)	A promising tool for the rapid assessment of HL in a busy clinical practice to screen for potential literacy problems.	Only measures one dimension of HL. Presence of a ceiling effect. Does not measure the patient's understanding of the words.
HLQ	A nine-factor model was fit using 44 final items with no cross-loadings or correlated residuals. The fit was satisfactory CFI = 0.936 = 0.930, RMSEA = 0.076, and WRMR = 1.698. Correlations between factors showed a clear distinction between the agree/disagree scales, but less distinction for cannot do/very easy scales.	It measures multiple domains of HL.	Self-assessment that has a potential for self-report bias.
DNT-15	Correlated with REALM (0.54), WRAT (0.62), and Diabetes Knowledge Test (0.71); Internal reliability (0.95). It has good internal reliability (0.90 and 0.89); split sample analysis, correlated with the full DNT in both subsamples (0.96 and 0.97).	Tests numeracy is associated with diabetes management.	Validated in highly educated sample and resulted in mean score correct of 61%—may be difficult or require high numeracy skills.

### Instrument Characteristics

The identified instruments used for assessing the level of HL and PTHL in extracted works are the following: Korean Functional Test HL (1 study), Newest Vital Sign, NVS (1 study), Functional, Communicative and Critical Health Literacy scale, FCCHL (1 study), Health Literacy Survey European Questionnaire 47, HLS-EU-47 (1 study), Test of Functional Health Literacy in Adults, TOFLHA (1 study), Test of Functional Health Literacy in Adults–Short Form, S-TOFHLA (8 studies), Rapid Estimate of Adult Literacy in Medicine–Revised, REALM-R (1 study), 3 brief screening questions, 3-brief SQ (6 studies), Rapid Estimate of Adult Literacy in Medicine, REALM (3 studies), Health Literacy Questionnaire, HLQ (1 study) and Diabetes Numeracy Test 15, DNT-15 (1 study) (37, 58–80). There are considerable differences found in their structure, number of items, administration time, available languages, type of administration, scoring system, measurement scope and properties, implicating their use in different settings.

The most used measures of HL in patients with type 2 diabetes mellitus were S-TOFHLA, then 3-brief SQ, REALM and HLS-EU-47. The S-TOFHLA, TOFHLA and REALM have been validated in different populations and are used in validation studies for 3-brief SQ and NVS. They are considered as a gold standard in validation studies. However, 3-brief SQ and NVS have a broader measurement scope and better properties, which put them in a better position for use in future validation studies. S-TOFHLA, REALM and HLS-EU-47 are not practical in busy clinical settings and REALM requires researcher participation. While the most used instruments, S-TOFHLA and REALM, measure only the functional domain of HL, the others 3-brief SQ, and HLQ address functional and critical HL, and DNT-15, KHLS and TOFHLA functional HL and numeracy. The only one for examination of all three levels of HL individually, their mutual correlation and different effects on patient is FCCHL.

### Validation, Strengths, and Limitations

NVS - Good reliability and convergent validity with well-validated and commonly used measures of HL such as the TOFHLA. Strengths are related to its suitability for rapid assessment of low HL. Test format might intimidate respondents.

FCCHL - Strong positive evidence for its content and structural validity and moderate positive evidence for internal consistency. This scale includes three levels of HL, each of which might have different effects on patient outcomes. It is proved to be easy to administer in a clinical setting. The scale is not validated in English.

Three-brief SQ - Positive evidence for the criterion validity of the 3-SQ with the S-TOFHLA (36 items) and limited negative evidence for its hypothesis testing validity and internal consistency. Instrument is validated in several diverse sample populations. It is quick, easy, and inexpensive for administration. Limitation is related to self-assessment and potential for self-report bias.

HLS-EU-47 - High levels of internal consistency reliability. It is available in many languages, length of assessment increases response burden.

S-TOFHLA - Demonstrated evidence for the internal consistency due to there being no evidence of structural validity. It has been validated in several diverse populations. Lack of this instrument is that it may not capture an individuals' HL in the dimension of numeracy.

REALM - Found to have a high test-retest reliability of 0.99. It is assessed by healthcare professionals and was found to have good face validity; however, it lacks in construct validity. It is quick and easy for administration, limited to the ability to pronounce words without being able to measure the patient's ability to understand the instructions on labelling of prescribed drug.

HLQ - Positive moderate evidence for its content validity and internal consistency and unknown evidence for structural validity. Strength is that measures multiple domains of HL, but due to self-assessment has a potential for self-report bias.

DNT - Moderate evidence for its content validity and internal consistency and limited positive evidence for structural validity. This is test numeracy that is associated with diabetes management. Limitation is that can be difficult or require high numeracy skills (49, 78, 81–89).

### Health Literacy and Pharmacotherapy Literacy

Preliminary data in relation to HL and PTHL were extracted from the studies included in the qualitative synthesis and summarized in Table 6 (for HL) and Table 7 (for PTHL).

**Table 6:** Basic characteristics of the included studies for HL1.

**Instrument**	**Coutry**	**Sample size**	**Results**	**References**
S-TOFHLA	USA	1,002	HL levels have not been associated with glycemic control or the health consequences of type 2 diabetes	(80)
REALM	USA	398	HL levels were not associated with HbA1C blood levels	(79)
FCCHL	Japan	138	The three HL scales were only moderately correlated with each other, suggesting that each represents a different domain of HL abilities and skills.	(78)
KHLS	South Korea	103	71.7% of patients had limited HL	(77)
S-TOFHLA	USA	189	HL was not associated with blood glucose measurement, but was associated with recording of glucose measurement	(76)
3-brief SQ	USA	14,102	Patients with limited HL were less likely to log on to the patient portal	(75)
TOFHLA	USA	102	36.3% of patients had limited HL	(74)
S-TOFHLA	USA	250	The level of HL has not been linked to the use of the Internet	(72)
S-TOFHLA (Spanish)	Spain	144	46.5% of patients had limited HL	(70)
3-brief SQ	USA	1,366	72% of patients had limited HL	(69)
3-brief SQ	Canada	154	Limited HL has been observed in patients with type 2 diabetes who have also been diagnosed with depression	(68)
3-brief SQ	Netherlands	1,714	Lower HL was significantly associated with less diabetes knowledge, higher HbA1c level, less self-control of glucose level, and less physical activity	(67)
3-brief SQ (1 question)	Switzerland	493	8.7% of patients had limited HL	(66)
S-TOFHLA	USA	288	32.8% of patients had limited HL	(65)
3-brief SQ	Canada	1,948	12.6% of patients had limited HL	(64)
HLQ	Denmark	46,354 of which 1,685 participants were diagnosed with diabetes	Even after adjusting socio-demographic characteristics, people with diabetes and limited HL were more likely to be physically inactive and had unhealthy eating habits compared to people with high levels of HL	(63)
NVS	Taiwan	232	76.3% of patients had limited HL	(62)
HLS-EU-Q47 FCCHL	Norway	388	Good general health, education and empowerment were positively associated with HL in people with T2DM. They explained about 17% of the total variance in HL	(61)
S-TOFHLA	Brazil	347	A significant number of patients did not have adequate HL	(60)
S-TOFHLA	Iraq	280	Most subjects had limited HL and poor glycemic control	(59)

**Table 7:** Basic characteristics of the included studies for pharmacotherapy literacy.

**Instrument**	**Coutry**	**Sample size**	**Results**	**References**
S-TOHFLA DNT-15	Hungary	102	34.6% of the patients with T2DM had inadequate/marginal reading and comprehension level	^*^(58)
REALM	USA	383	The level of HL has been linked to the effectiveness of patient treatment	(37)
REALM-R	USA	125	HL levels have been linked to the level of diabetes knowledge, but have not been linked to glycaemia or medication	(73)
REALM	USA	50	The level of HL has been linked to level of knowledge about diabetes	(71)

* *Accessing both–health and pharmacotherapy literacy*.

In the research period most of the studies were published in the period from 2006 to 2021 (37, 58–80). The largest number of studies was conducted in the United States (11 studies) and Canada (2 studies). In contrast, in South Korea, Japan, Switzerland, Taiwan, Spain, Norway, Brazil, Denmark, Iraq, Netherlands and Hungary, one study was performed. Cross-sectional studies make up the majority (20 studies), while three are longitudinal and one is a cohort study. The sample size ranged from at least 50 to 46,354 subjects and are adults over the age of (37, 58–80).

In six studies, limited HL was observed in <50% of subjects, and in five studies, more than 50% of subjects had limited HL. HL levels have been linked to diabetes knowledge and treatment efficacy, while HbA1C concentrations, Internet use, glycemic control, and health consequences have not been linked to HL levels (37, 58–80). One study found an increased prevalence of people with limited HL who were diagnosed with depression (67), while another study found that people with limited HL were more likely to eat unhealthily and had reduced physical activity compared to people with high HL (62).

## Discussion

This work presents the most comprehensive inventory of HL and PTHL measures in patients with type 2 diabetes mellites to date. There are limited number of works assessed the instruments that measure HL in patients with type 2 diabetes and they were focused only on available self-administered instruments in regards of validation aspects (90), however this work presents the broader perspective including the more comprehensive report on their structure, measurement scope, scoring etc. allowing possibilities for clinicians, health professionals and researchers to evaluate available HL and PTHL instruments and match them with the goals of their work.

HL has been presented as a measurable and important concept in considering education for patients with chronic diseases such as diabetes. It has been shown that in comparation to the other scales that focus exclusively on functional HL, FCCHL covers all three levels of HL, each of which can have different effects on patient outcomes. Also, the scale is easy to apply in clinical conditions (61).

The identified instruments have inherent strengths and weaknesses as a result of their structure, properties and measurement scope. The REALM, NVS, TOFHLA/ s-TOFHLA, DNT, KHLS, FCCHL and NVS are designed to directly measure specific skills and have some limitations in administration, especially in clinical settings where they are more likely to cause anxiety and shame among patients with inadequate HL and PTHL skills. Self-administrated instruments such as 3-brief SQ, HLS-EU-47 and HLQ non-directly measure certain skills and they are less likely to cause the anxiety and shame which makes them more suitable to be used in clinical settings and research applications. Many self-reported measures are designed as screening tests that may be differentially sensitive and specific than measures developed to more fully describe HL for research or clinical purposes. This is also seen in other articles (21, 91, 92).

The NVS had good sensitivity and may be more sensitive than the TOFHLA for marginal HL (83). Using the test can alert physicians and pharmacists to focus on the patients who require more attention and help them communicate with those patients by using recommended techniques.

The REALM and the TOFLHA focus primarily on reading-related skills and therefore do not present comprehensive measures for the skills needed by individuals in the healthcare system (21).

Time of administration plays a significant role in clinical settings. In this regard 3-brief SQ, NVS, FCCHL, REALM, REALM-R, S-TOFHLA and HLQ are relatively quick and easy for an administration and can be considered in different clinical settings and survey researches.

The type of administration must also be considered for practicality in clinical settings. REALM, REALM-R, KHLS and DNT-15 require involvement of the researcher and could cause shame and discomfort. Since self-administered instruments TOFHLA, S-TOFHLA, NVS, HLS-EU-47 and HLQ are very unlikely to cause discomfort, they require good visual abilities, full concentration, and good writing skills. Three-brief SQ and FCCHL can be administered in both ways and are more flexible and convenient for use.

A lack of researches with PTHL questionnaires in patients with type 2 diabetes mellitus is a very limiting factor for this population since the use of multiple drugs/insulins is very common. REALM only measures one dimension of HL and does not assess the patient's understanding of the words. DNT-15 is the test for numeracy.

The findings of this review can be used for other chronic conditions with similar HL and PTHL demands on individuals. This review did not only address the usefulness and applicability of the instruments in individuals with diabetes but also provided an evaluation of these instruments and their strengths and weaknesses, which are transferable for evaluating their applicability in other health conditions and situations.

### Practice Implications

As instruments for measuring HL and PTHL continue to be published, authors advise clinicians, health professionals and researchers to evaluate available HL and PTHL measurements for a conceptual and practical match with the goals of their work.

When choosing a practical match, style of administration, purpose for measurement, their basic characteristics: domains, methods of assignment, structure, method of scoring, validation, strengths and limitations should be considered. It is important to align with the topic or task under consideration and choose the one that has been validated in a similar target population in order to have an accurate measure of the domain being assessed. Predictive qualities and appropriateness for assessment of changes in HL and outcomes over time have to be taken under consideration.

FCCHL was evaluated as the most appropriate instrument to apply to people with diabetes since a diabetes-specific type of instrument and the contents of its items may be more sensitive in a diabetes clinical setting targeted at diabetes, it is a model-based and comprehensive measure which covers all 3 levels of HL and the evidence for the measurement properties are better than those for the other instruments. However, his structural validity needs to be further established, and therefore adding DNT-15 questionnaire can be one of the options for considering application of FCCHL in this population (90).

Based on the previous considerations FCCHL and 3-brief SQ have the broadest measurement scopes. They are quick, easy, and inexpensive for administration. Three-level HL can be considered as the most useful and comprehensive instrument to screen for inadequate HL. The limitation is that the English version is not validated. Three-brief SQ has many advantages in comparison to other instruments, including that it is less likely to cause anxiety and shame. This instrument can be considered the best for measuring functional HL in patients with diabetes mellitus type 2 and other chronic diseases.

### Limitations

This review was subject to some limitations. The use of non-interventional studies, the heterogeneity of studies in terms of samples represent important limitations for this scoping review. Only works written in English have been considered. Assessments of the instruments' dimensions, strengths, and limitations were made on the basis of our own experience and judgment; and as such, this was a subjective review. In order to minimize the effect of the issue of subjectivity, each measure was analyzed by multiple authors, and any discrepancy was addressed by all the authors and resolved through fruitful discussion.

## Conclusion

The ongoing development of instruments suggests that there is still a need for comprehensive measurement across diverse populations. Three-brief SQ has been found convenient for use in populations with diabetes mellitus type 2 taking into consideration the broadest measurement scope, demonstrated good measurement properties, that has many advantages over other instruments, and could be considered the best available instrument to measure functional HL. FCCHL scale measures the broader concept of HL, including the ability to retrieve, understand, and use health-related information and could be one of the most appropriate and comprehensive instrument for measuring HL in people with diabetes. However, it has not been validated in English and the future research must be directed in this way, as well as establishing of the structural validity of the questionnaire.

The results of the studies show that HL may be directly related to the clinical outcome in patients with diabetes and that each individual level of HL could act differently. The ways in which each level of HL influences patient behaviour about care and health outcomes should be further explored.

So far, PTHL questionnaires (REALM/R and DNT-15) have not found their best application in people with type 2 diabetes mellitus and further research should certainly be aimed at developing a specific PTHL questionnaire for people with type 2 diabetes mellitus, due to the nature of the disease itself and the frequent use of multiple drugs in its therapy.

This works provides information to enable practitioners, health professionals and researches to select the most appropriate instrument available for measuring HL and PTHL in patients with type 2 diabetes mellitus.

## Data Availability Statement

The original contributions presented in the study are included in the article/supplementary material, further inquiries can be directed to the corresponding author/s.

## Author Contributions

DK and ML contributed substantially to the conception, design of the study, screening, and selection of the studies. ML produced the first draft of the paper, which was extensively redrafted by DK, with significant input from NB-S. All authors contributed to the interpretation of data and read and approved the final manuscript.

## Funding

The research of DK and NB-S was partially funded by the Ministry of Education, Science and Technological Development, Republic of Serbia through Grant Agreement with University of Belgrade-Faculty of Pharmacy No: 451-03-9/2021-14/200161.

## Conflict of Interest

The authors declare that the research was conducted in the absence of any commercial or financial relationships that could be construed as a potential conflict of interest. DK and NB-S are also co-authors of one paper reviewed in this study. However, the authors believe that the systematic inquiry undertaken in this paper is transparent, reproducible, and sufficiently neutral.

## Publisher's Note

All claims expressed in this article are solely those of the authors and do not necessarily represent those of their affiliated organizations, or those of the publisher, the editors and the reviewers. Any product that may be evaluated in this article, or claim that may be made by its manufacturer, is not guaranteed or endorsed by the publisher.
